# An efficient edge/cloud medical system for rapid detection of level of consciousness in emergency medicine based on explainable machine learning models

**DOI:** 10.1007/s00521-023-08258-w

**Published:** 2023-03-15

**Authors:** Nora El-Rashidy, Ahmed Sedik, Ali I. Siam, Zainab H. Ali

**Affiliations:** 1 Department of Machine Learning and Information Retrieval, Faculty of Artificial Intelligence, Kafrelsheiksh University, Kafr El-Sheikh, Egypt; 2 Smart Systems Engineering Laboratory, College of Engineering, Prince Sultan University, 11586 Riyadh, Saudi Arabia; 3 Department of the Robotics and Intelligent Machines, Faculty of Artificial Intelligence, Kafrelsheiksh University, Kafr El-Sheikh, Egypt; 4 Department of Embedded Network Systems Technology, Faculty of Artificial Intelligence, Kafrelsheiksh University, Kafr El-Sheikh, Egypt

**Keywords:** Emergency medicine, Explainability, GCS, Machine learning, Edge/cloud medical system

## Abstract

Emergency medicine (EM) is one of the attractive research fields in which researchers investigate their efforts to diagnose and treat unforeseen illnesses or injuries. There are many tests and observations are involved in EM. Detection of the level of consciousness is one of these observations, which can be detected using several methods. Among these methods, the automatic estimation of the Glasgow coma scale (GCS) is studied in this paper. The GCS is a medical score used to describe a patient’s level of consciousness. This type of scoring system requires medical examination that may not be available with the shortage of the medical expert. Therefore, the automatic medical calculation for a patient’s level of consciousness is highly needed. Artificial intelligence has been deployed in several applications and appears to have a high performance regarding providing automatic solutions. The main objective of this work is to introduce the edge/cloud system to improve the efficiency of the consciousness measurement through efficient local data processing. Moreover, an efficient machine learning (ML) model to predict the level of consciousness of a certain patient based on the patient’s demographic, vital signs, and laboratory tests is proposed, as well as maintaining the explainability issue using Shapley additive explanations (SHAP) that provides natural language explanation in a form that helps the medical expert to understand the final prediction. The developed ML model is validated using vital signs and laboratory tests extracted from the MIMIC III dataset, and it achieves superior performance (mean absolute error (MAE) = 0.269, mean square error (MSE) = 0.625, *R*^2^ score = 0.964). The resulting model is accurate, medically intuitive, and trustworthy.

## Introduction

Emergency medicine (EM) is a rapid-growing specialty which is critical and important for the society. The patients are received in urgent cases in which rapid tests and evaluation of vital signs are very important to obtain an accurate diagnosis and make decisions. Therefore, this field attracts researchers to investigate solutions in it. Artificial intelligence is strongly involved in these investigations including machine learning (ML) and deep learning (DL) [1]. One of the important medical issues is the detection of level of consciousness which is considered as one of the important observations of the patient. For this reason, the care givers should rapidly handle the patients in this case to survive them [2].

Level of consciousness can be obtained by evaluation of Glasgow coma scale (GCS) using several methods such as electroencephalography (EEG) and vital signs [3, 4]. Traditionally, the consciousness of a certain patient can be determined based on his eye opening, verbal and motor responses which are the factors of GCS. It is a dominant method which is scaled from 0 to 15. This method needs medical examination from the medical expert which is not available all the time. Therefore, there is a need to investigate an automatic method for patients based on vital signs such as hypotensive, heart-rate-disordered and hyperthermal patients and laboratory tests such as albumin and hemoglobin in addition to collecting some medical records such as EEG and EPG signals [5].

The field of artificial intelligence is involved in Big Data and Analytics [6], Cloud/Edge Computing-based Big Computing [7] and the Internet of Things (IoT)/Cyber-Physical Systems (CPS) [8, 9] applications. These applications dominate industry and research for the development of various smart-world systems [10, 11]. Large, complicated datasets may now be approximated and reduced into extremely accurate predictions and transformative output using artificial intelligence, making human-centered smart systems much easier to implement [12–14]. Machine learning techniques can be used to any types of data such as visual, auditory, numerical, text or some combination [15]. Therefore, engineers can build their edge-based platforms based on machine learning techniques due to its high performance and low time consumption. Furthermore, the deployment of machine learning algorithms is involved to providing a security environment to ensure the privacy of the data of the patients which is transmitted through the network [16, 17]. Therefore, this study comprises the issue of security and privacy and its importance in the edge communication system [18].

The utilization of ML in medicine has witnessed an explosion in numerous medical applications [19–22], including automated diagnosis, classification of disease severity, development of new therapies [23], analysis of medical record [24] and improving the quality of medical data [25]. The use of ML methods in automated diagnosis has bifurcated in diverse disease types, including corona virus [26–30], kidney disease [31, 32], heart disease [33, 34], cancer [35, 36], diabetes and retinopathy [37, 38], skin lesion [39] and other diseases [40–42].

• Motivation and contributions

The GCS has been extensively used to objectively describe the extent of impaired consciousness within all types of acute medical and trauma patients. GCS assesses patients based on the following aspects: (i) eye-opening verbal, (ii) motor and (iii) verbal responses. These scoring factors are not obtained automatically, which may cause less detailed description. In addition, there is a need to overcome the difficulty with early detection and diagnosis. Therefore, the objective is to provide a numerical method to evaluate the GCS accurately has become indispensable. The main objective of this work is to introduce a machine learning-based system that is performed through IoT and edge/cloud system to enable automatically measuring the level of consciousness. The proposed system consists of three main phases: (i) vital signs acquisition, (ii) Fog-Assisted Consciousness Management (FACM) and (iii) cloud server and clinical service delivery model. In addition, the proposed system is carried out on the MIMIC dataset. Furthermore, the proposed model maintains the interoperability issue by providing natural language explanation for the developed decisions, in order to provide answers for the medical straightforward inquiries. Contributions of this paper can be illustrated in the following points:

1. Investigate a new method for GCS evaluation for automatic estimation of the resulting score.
2. Build an internet of medical things (IoMT) system through edge/cloud technology.
3. Deploy machine learning techniques for detection of level of consciousness.
4. Maintain the interoperability of the ML model in order to provide explanation to the outcome of the model.
5. Compare the deployed techniques to obtain an optimal one in terms of evaluation metrics.
6. Recommend an optimal system and discuss the limitation of its application.
7. Evaluate the effectiveness of adopting the fog technology on the proposed system.

This paper is organized as follows: In Sect.  2, background and related work is reviewed. In Sect. 3, an edge/cloud system for consciousness detection is proposed. In Sect. 4, experimental results are detailed. Section 5 presents a discussion for the results and comparison with the works in the literature. In Sect. 6, the paper is concluded.

## Background and related work

This work proposes an edge/cloud medical system whose objective is to estimate the level of consciousness. For this purpose, we deployed a set of ML techniques to predict the value of GCS automatically. This section discusses the works in the literature which are relevant to the proposed system. Firstly, we discuss the GCS. GCS is one of the most utilized scores for responsiveness assessment of inpatients. It was introduced in 1974 to standardize the clinical assessment of level of consciousness in patients with head injuries [43]. GCS is an effective means to compare responses of patients in different coma states and to compare effectiveness of treatments [44]. GCS is a realization of three components: motor response, verbal response and eye opening. The scale originally consisted of fourteen points, four for eye opening and five for each motor response and verbal response. A sixth point was added 2 years later for motor response [45]. The GCS and its score points are shown in Table 1.

**Table 1:** Glasgow coma scale

Behavior	Response	Score
Eye opening	Spontaneous	4
	To speech	3
	To pain	2
	No response	1
Verbal response	Oriented	5
	Confused conversation	4
	Inappropriate words	3
	Incomprehensible sounds	2
	No response	1
Motor response	Obeys commands	6
	Localizes pain	5
	Flex to withdraw from pain	4
	Abnormal flexion	3
	Abnormal extension	2
	No response	1

The manual calculation of GCS score involves summing the scores corresponding to the best response for each individual behavior. Hence, the total score has values between 3, being in deep coma or death, and 15, being fully alert.

To the best of our knowledge, there are no relevant contributions in the literature that exploit laboratory tests and vital signals to automatically provide a numeric estimate of GCS level using machine learning techniques. Consequently, this study is the first to conduct a similar method and results. However, early studies have shown the feasibility of using GCS levels to automatically determine the functional state of the autonomic nervous system (ANS) in coma patients. However, these studies classify the consciousness level into subgroups: two subgroups (with GCS from 3 to 5 and from 6 to 8) [46], or three subgroups (low, mild and high consciousness) [4].

Estévez et al. [46] presented an approach to classify coma patients into two subgroups according to their GCS based on the heart rate variability (HRV). The experiments were conducted on 47 patients in coma. All patients were in ICU and mechanically ventilated. In this approach, ECG signals have been extracted, resampled into 1000 Hz and then processed using Hilbert–Huang transform (HHT) to extract a number of key spectral features. A logistic regression model was implemented to classify the consciousness level of patients into two categories: deep and mild coma, based on their HRV. They reported that their model achieved an overall efficiency of 95.74%.

Latifoğlu et al. [4] proposed an approach for automatic evaluation of the state of consciousness of coma patients based on EEG signals. The state of consciousness is classified into either low, mild or high based on GCS levels. They obtained EEG signals from 34 coma patients in ICU. Features are extracted using power spectral density (PSD) method. The authors adopted various machine learning classifiers to classify the consciousness level, and they obtained an accuracy of 92.5%.

Furthermore, machine learning algorithms are widely adopted in health care, relying on medical data to predict or classify various health states [47, 48]. Also, ML and DL have wide applications in emergency medicine [49–53]. An ML-based model was presented in [54] to predict the outcome of patients after traumatic brain injury (TBI). GCS level, besides the other thirteen parameters, was involved in predicting the patients’ outcome. Authors have conducted a performance comparison of different nine ML algorithms and reported that the random forest algorithm had achieved the best performance in outcome prediction with an accuracy of 91.7%. They also concluded that GCS score, besides age, fibrin/fibrinogen degradation products and glucose are the most important factors for outcome prediction. Tsiklidis et al. [55] implemented an ML model based on a gradient boosting classifier to predict the mortality rate of trauma patients at admission. They relied on the GCS and other seven health parameters to train the model. The accuracy of the model was 92.4%. The authors remarked that GCS, age and systolic blood pressure had the highest impact on the final decision of the model.

In addition, Hall et al. [56] implemented a decision tree-based model to identify patients with a potentially modifiable outcome after intracerebral hemorrhage (ICH). They demonstrated that the GCS score is one of the most important predictors to identify the patient outcome. Similar results were concluded in [57] and [58] as the GCS score is the most significant variable in predicting the outcome and mortality of TBI, and subarachnoid hemorrhage (SAH) patients, respectively, using various machine learning models [59, 60].

The term "fog computing" refers to a paradigm that brings cloud computing and its associated services to the edge of a network. In this manner, various issues that are inherently associated with cloud computing, such as latency, lack of mobility support and lack of location awareness, are solved [61]. Fog computing and cloud computing have common and distinct features [62, 63]; Table 2 lists a comparison between the fog and cloud platforms in terms of various technical aspects. With the existence of IoT devices, the requirements for high bandwidth, security and low-latency applications are raised [64]. Therefore, fog computing is fitted here to provide such requirements for IoT networks. Recently, ML has been widely adopted with fog computing to enhance its services. Abdulkareem et al. [61] investigated the different roles of ML in the fog computing paradigm and provided diverse improvements in ML techniques associated with fog computing services, such as security, accuracy and resource management. Kishor et al. [65] presented an ML-based fog computing approach to minimize latency in healthcare applications. They implemented a multimedia data segregation scheme in fog computing to reduce the total latency resulting from the data transmission, computation and network delay. They employed the random forest model to calculate the total latency. The reported results reveal that 95% reduction in latency is achieved compared to another pre-existing model. Khater et al. [66] proposed a lightweight intrusion detection system (IDS) for fog computing using a multilayer perceptron (MLP) model. They evaluated the developed system against two benchmark datasets: Australian Defense Force Academy Linux Dataset (ADFA-LD) and Australian Defense Force Academy Windows Dataset (ADFA-WD), which contain exploits and attacks on various applications. The developed system is implemented using a single hidden layer, and it achieved a 94% accuracy in ADFA-LD and 74% accuracy in ADFA-WD.

**Table 2:** A comparison between the old challenges that caused by cloud computing and the added updates using fog computing and SDN

Parameter	Cloud computing	Fog & SDN
Processing operation [62, 67, 68]	Very high	Moderate
Data processing [67, 68, 69]	At cloud server	Locally—at fog and SDN
Transmission delay [62, 70, 68]	High	Very low
Location-awareness [67, 68, 70]	No	High
System reliability [67, 68, 69, 71]	Support	Support
System scalability [62, 67, 68]	Support	Support
Geographical distribution [62, 67, 68]	No	High
Real-time interactions [68]	Not fully support	Support
Power consumption [68]	High	Low
Ubiquitous services [68]	Support	Support
Management model [68]	Centralized	Decentralized
Decision-making [68]	Remotely	Locally
Security [68]	It’s hard	Easy to apply and maintain

## Proposed edge/cloud system

This work proposes an edge/cloud system for consciousness detection. The deployed scenario is based on fog-assigned consciousness managing system. The main objective of this proposed system is to enable the scoring system based on vital signs and laboratory tests such as blood pressure, heart rate, respiratory rate and oxygen flow rate to determine the consciousness level of the patient. A theoretical approach is first introduced. Recent research in describing the subsequent level of consciousness has been concerned with determining GCS, which includes several functions such as eye-opening, verbal response and motor response. Then, the collected information is recorded manually by a therapist in a local system computer-based or paper-based. In some cases, this method used may ignore significant factors such as alcohol intoxication, low blood oxygen and drug use histories. In addition, it may suffer a delay time in decision making. All these issues lead to an inappropriate score that can alter and negatively affect a patient’s level of consciousness. Therefore, introducing a dynamic data exchange environment with a high ability to deal transparently with a large scale of vital functions should be required.

**Fig. 1:**
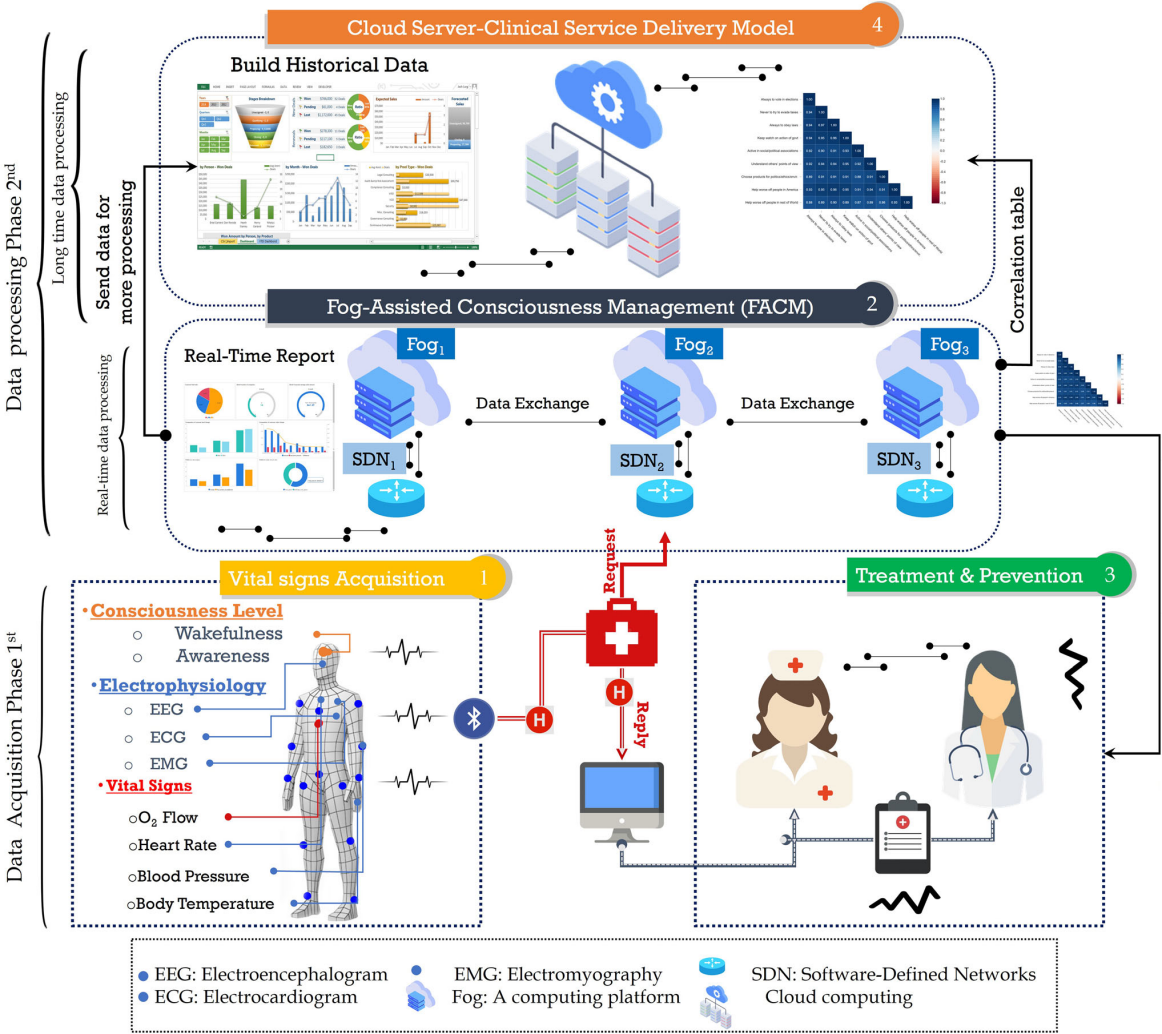
Conceptual design of the edge/cloud system

The structure of the proposed edge/cloud system, as shown in Fig. 1, consists of three main phases: (i) vital signs acquisition, (ii) Fog-Assisted Consciousness Management (FACM), and (iii) cloud server and clinical service delivery model. The edge/cloud system can be implemented in a wide range of intelligent healthcare sectors that are characterized by a massive infusion of data and a high need for careful and rapid decision-making, such as emergency departments and intensive care units.

### Vital signs acquisition

The operation in this face divides into two directions are: (i) collecting information of vital signs from the real sensors that directly connected to the patient; and (ii) getting information from the medical report prepared by specialists. Lately, IoT sensor devices play an essential role in medical construction in which it provides interactive real-time network connection to the users and medical devices through various communication technologies. Wearable devices are a part of IoT devices that allow sensor devices to collect real information from patients about their vital signs from everywhere at any time; this technique is called ubiquitous technology. The cope of these collected vital signs will be through the software-defined network (SDN) and fog computing installed in FACM layer. In the traditional architecture proposed in several types of research, the data will transfer to the cloud server; this technique affects not only network bandwidth but also response time. Table 2 describes old challenges that caused by cloud computing and the added updates using fog computing and SDN.

### Fog-Assisted Consciousness Management (FACM)

This second phase includes several operations related to managing the process of data transmission from and to the cloud server and end-user within the treatment and prevention phase. The integration between SDN and fog technology initially enables data communication systems to be more dynamic, secure, and reliable. The security and data reliability achieved by the SDN will establish a private channel with the fog server via its controller in OpenFlow to ensure the level of data privacy. Inside this channel, the fog server applies an access control policy that is predefined by the fog setting. For more data protection, the fog server uses the integrity check process via adding octets/bytes with every data packet sent [62]. This technique is a common Internet technique that is used to notify the fog server about any change in the bytes of data. The operations in this phase are listed as follows:

- Providing a decentralized data transmission strategy through distributed SDN nodes integrated with other deployed fog nodes. This strategy supports local data processing and avoids unnecessary data traveling to the cloud server, thus reducing network bandwidth usage.
- Establishing a secure channel for boosting data protection during the operation of data exchange between SDN and IoT sensors and SDN and fog nodes.
- According to vital signs that sent from the lower layer, the fog computing starts to build a predictive model of consciousness level based on an unsupervised learning technique called the principal component analysis (PCA).

**Fig. 2:**
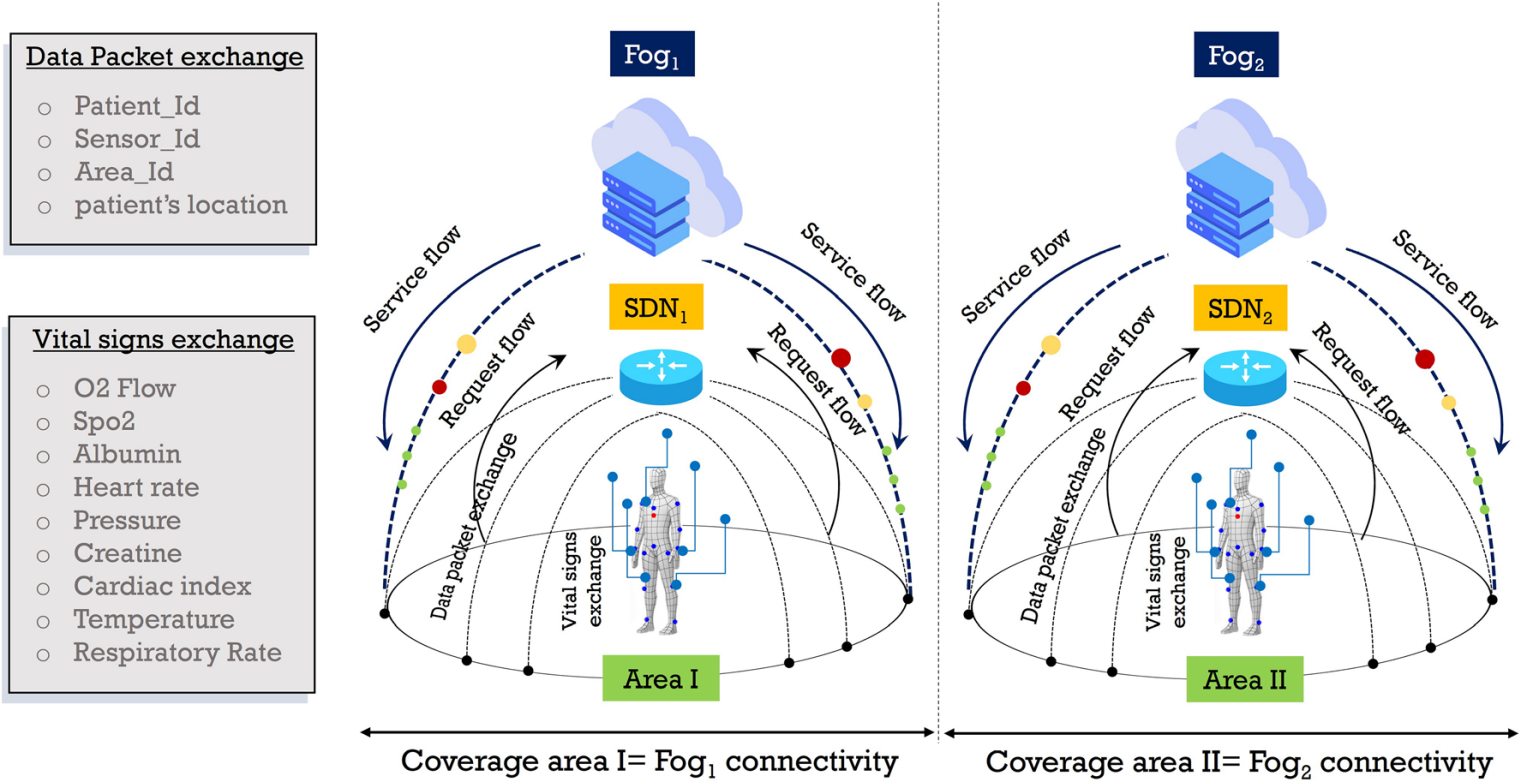
The process of data and vital signs exchange from patient to SDN and from SDN to Fog

As shown in Fig. 2, the vital signs are sent from sensors in an event shape. Each event includes sensor_ID, patient_ID, patient’s location, flow entries that tell the SDN what to do with an incoming packet, as well as the patient’s vital signs. Using OpenFlow (OF) protocol, the events will convert to flow entries for feeding SDN. This technique increases SDN capability in network monitoring and management.

From the network point of view, achieving better network connectivity relies on avoiding communication range violation during the process of data transmission [62]. Therefore, the available communication range for each SDN is determining according to the available predefined range assigned by the fog node. For instance, as seen in Fig. 2, the communication range in coverage area I is determined by Fog_1_ and thus the SDN_1_ can only direct connected with sensors deployed in this area. Each event sent from this area has been addressed by a labeled information about sensors and patients connected in this area only. In addition, SDN1 has equipped with cache memory for saving requests and adding security rules for them. Table 3 illustrates a sample of data stored in the SDN_1_ caching table.

**Table 3:** A sample of data stored in the SDN_1_ caching table

Header of data packet							Vital signs					
Area_ID	Sen_ID	Flow entry#	Patient_ID	loc		Pub. Key	Priv. Key	O_2_ Flow	HR	PR	RR	BUN
				X	Y							
I-2	Srl | 186	123	PHU-14708	52	12	AABCID234781cy2E48v	EDOLWIFI2eMYdf5421T	80–100	60–100	70/120	12–16	6–24

The next operation in SDN is between SDN and Fog node. Each SDN generates its own private and public key for adding them to the transferred data to the fog node. For example, a single fog node can manage multiple SDNs in the same area or even different areas to provide a large scale of data processing and analysis and overcome high response time. Herein, the data in the fog node have become accessible from multiple users, and it needs to protect.

The transferred data from the SDN to the fog node will be addressed by some flow entries and SDN_ID for labelling. Moreover, the SDN adds a public key with each packet for protection. Only authorized fog node has the private key to decode this packet. It is worth to point out that only data related to the packet remain confidential, while vital signs are sent without encryption for ensuring the acceleration of fog performance. Table 4 discusses the steps of data transmission between SDN and fog node.

**Table 4:** The steps of data transmission between SDN and fog node

Step	Description
1	Each SDN stores sensing data received from its coverage area into a flow table. This table contains two types of the information: (i) information about forwarded data packet and (ii) information about vital signs
2	Each SDN generates its own public key and adds it to every data packet as it is shown in Table 3
3	The traffics between SDN and Fog node in the same area are occurred in a secure channel using SSH protocol
4	SDN uses a public key to encrypt only the header of the packet, and therefore the data packet has become anonymous or without personally identifiable information. The vital signs only will send clearly
5	The traffic sent from SDN is directly connected to the fog node located in the same area. This process helps to mitigate communication overhead
6	Each fog node provides an intelligent model that can predict the consciousness level according to vital signs sent from SDN. Only fog node that has the private key can decrypt messages sent from a certain SDN
7	Fog in a certain area can send the data packet to another fog node in another area for processing. This technique helps to achieve network load balancing so that if the fog node is busy the message will immediately redirect to another fog node. In our case, the fog node only can communicate with other fog nodes in different areas, while each SDN can establish the communication between its fog node located in the same area

**Fig. 3:**
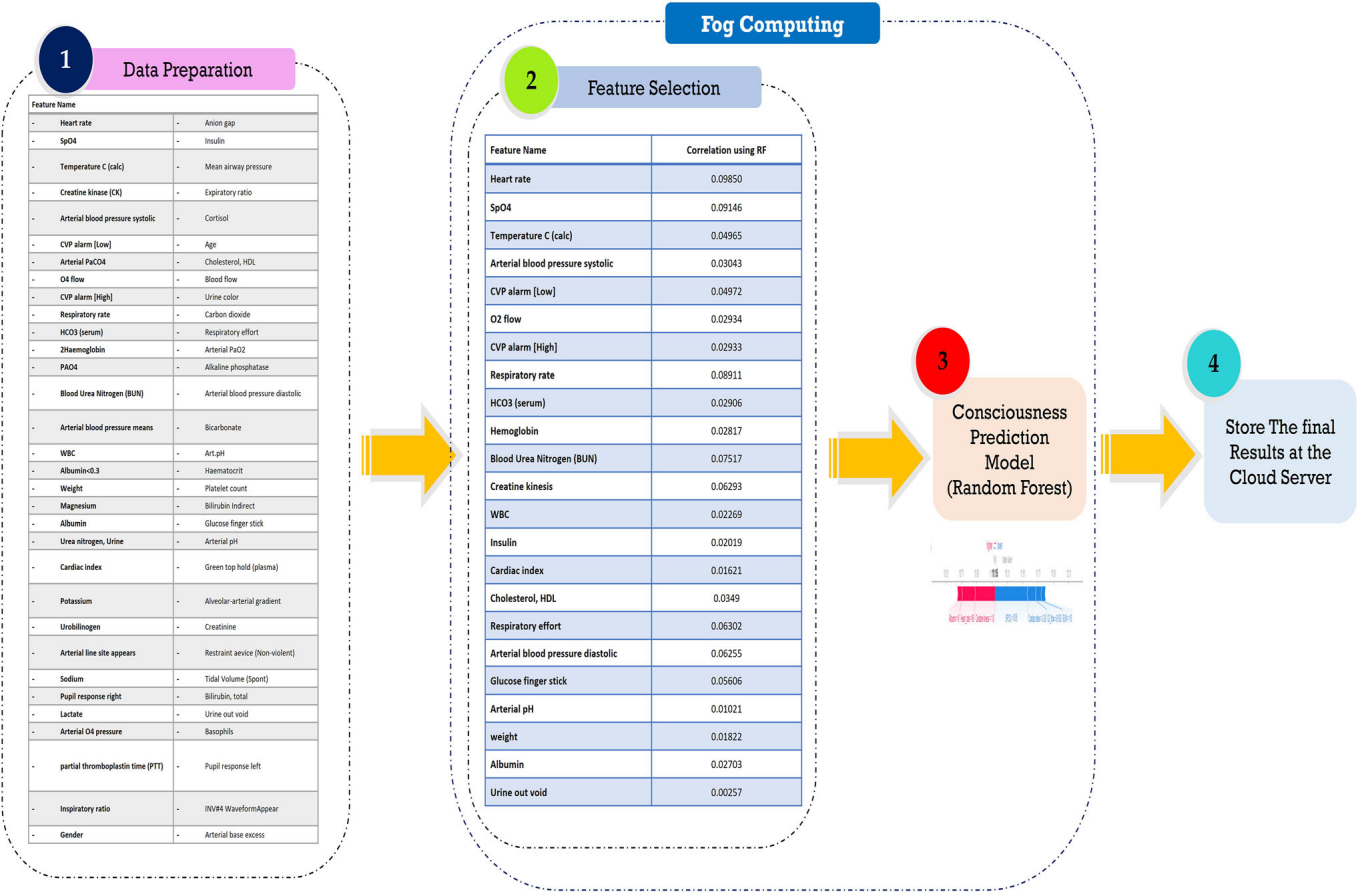
The main steps for calculating the level of consciousness

According to the proposed edge/cloud system, the deployment of fog nodes in the 2nd layer between the cloud server and IoT sensor devices plays a significant role in carrying out many fundamental operations on the received data from the SDN before passing it to the cloud server layer. One of these operations is related to reducing the consumption of network bandwidth [1, 2]. Fog node performs the processing on received data locally. Therefore, the data does not need to travel to the cloud server for processing. On the other hand, the fog nodes use a tree-based approach for building an intelligent model calculating the scoring conscious based on the vital signs received by SDN. Figure 3 depicts the main steps for building an intelligent model on the fog node.

The fog node takes the data preparation file from the previous phase in the 1st step. In the 2nd step, the fog node applies PCA for selecting the most effective features among a huge number of vital signs collected from the patient. This operation decreases the size of data that affects the system performance. In the 3rd step, the fog node builds the consciousness prediction model based on a random forest (RF) algorithm. The use of an RF to predict the GCS using vital signs has returned to several reasons that are: (i) RF gives the ability to measure feature correlation for all features using the Gini index that indicates the impact of each feature in the model [72], (ii) RF compromising the explainability and accuracy issues. Generally, models that have a good performance in terms of classification accuracy as SVM and LDA are not able to provide a clear explanation about the output decision [73], whereas the most tree-based algorithms are very good explainability, but may not be the best algorithm in terms of performance [74], and (iii) RF is a tree-based algorithm that utilized several trees and then combined the final decision using a majority voting algorithm. Information gain is used to split points in each tree. As a result, outliers are ignored by most trees that make RF a more stable algorithm [75]. In the 4th step, the GCS with correlation table will be stored at the cloud server to build historical data about these patients.

### Cloud server and clinical service delivery model

In the healthcare industry, cloud computing plays a pivotal role in supporting the shift of conventional storage to the digitalization of healthcare data. The revised vital data collected from the FACM phase will be travelled to cloud computing for saving, computing and analyzing. This technique affects network bandwidth usage and accelerates cloud decisions. With cloud computing, a historical healthcare database will build to wrap up patients' data flowing from FACM. This database intends to create data linkages throughout the healthcare systems, irrespective of where the data originate or are stored. Moreover, cloud-based data analysis can prepare more personalized care reports for patients on an individual level, and thus several healthcare-related functions will be improved in terms of GCS evaluation and detection of the level of consciousness automatically.

## Experimental results

### Experiment 1

This section is going to measure the performance of the edge/cloud medical system-based fog technology compared to traditional monitoring system without fog technology. The NS2.35 is used to model the wireless sensor network and fog sensor with varying network range from 100 to 400 sensor, Table 5 shows all network settings. The sensor nodes connect to others via IEEE 802.11p/WAVE. Table 6 depicts the configuration of the IEEE 802.11p interface described as it is in [76], and fog nodes were reconfigured based on the reference guide called “*Fog hierarchical deployment model*” from OpenFog Reference Architecture [77].

**Table 5:** Network setting

Parameter	Value
Sensor range	100–400
Simulation time	1500 S
MAC protocol	IEEE 802.11p
Channel type	WirelessPhy
Energy model	Battery
Antenna model	OmniAntenna
Packet size	512 bytes
Traffic source	CBR

**Table 6:** IEEE80211.p interface configuration [76]

Parameter	Value
Channel	175
Bandwidth	20 MHz
Frequency	5.875 GHz
Antenna gain	2dBi
Setup TxPower	23/18 dBm
Receiver sensitivity	− 95.2 dBm

**Fig. 4:**
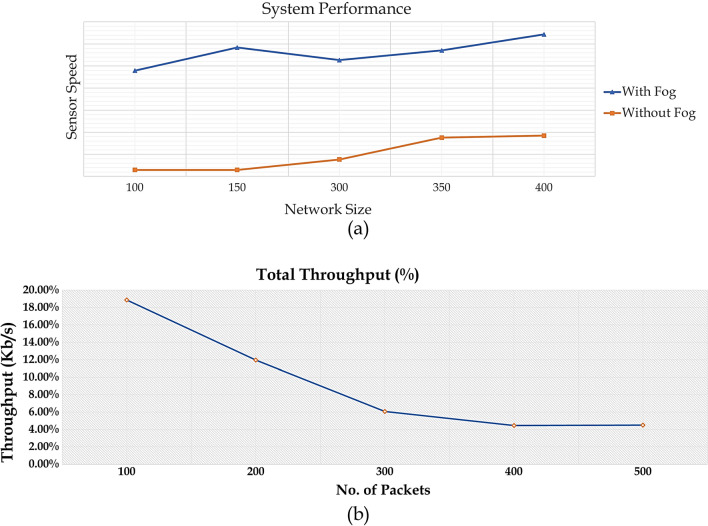
The performance of edge/cloud medical system-based on fog computing versus the traditional monitoring system-based on cloud computing is shown in figure (**a**), and the overall system throughput is shown in Figure (**b**)

Reliable communication has typically relied upon the quality of the packet transmission process. To this end, the performance of the proposed edge/cloud medical system in achieving high reliability was tested. In Fig. 4a, the *x*-axis denotes the network size, and the y-axis denotes the total speed of sensors, while in Fig. 4b, the *x*-axis denotes the total number of data packets, and the y-axis denotes the system throughput (kb/s). As observed shown in Fig. 4a b, the proposed system-based fog computing outperforms the traditional monitoring system-based cloud computing because the fog computing technology can increase the number of successful packets sent without affecting the network bandwidth, as is evident from Fig. 4b. Fog computing also provides the process of data processing locally, and thus, there is no need to transfer data via the Internet; only the data that need more processing and analysis will send to the cloud server.

### Experiment 2

In this experiment, we exploit the proposed edge/cloud system to improve the efficiency of the consciousness measurement through efficient local data processing. Moreover, an efficient machine learning (ML) model to predict the level of consciousness of a certain patient based on the patient’s demographic, vital signs and laboratory tests is proposed, as well as maintaining the explainability issue using Shapley additive explanations (SHAP) that provides natural language explanation in a form that helps the medical expert to understand the final prediction.

#### Data collection

Medical Information Mart for Intensive Care III (MIMIC-III) is a publicly available dataset for intensive care units (ICU). It comprises electronic health record (EHR) data extracted from the bedside monitors inside ICU units of the Israel Medical center in Boston, USA [78]. MIMIC III was approved and maintained by the Massachusetts Institute of Technology (MIT), and it is freely available on PhysioNet [79]. MIMIC III includes data for 46,520 different patients and 58,976 different admissions, gathered between 2001 and 2012 [80, 81]. Each patient is associated with minute-by-minute vital signs measurements and laboratory tests. The median age of adult patients is 65.8 years, and 55.9% of patients are males. The sampling interval of records ranges from few seconds to hours, according to the acquired physiological measurements.

In the current study, we extracted 10,349 records from the entire MIMIC dataset and are used to train/test the model to predict the level of consciousness. The data were randomly split into 80/20 ratio for training and testing the ML models, respectively. Appendix A gives some information (normal range, unit of measurement) of physiological measurements and laboratory tests that are adopted in the current study.

#### Data pre-processing

These steps aim to enhance the quality of the chosen dataset. MIMIC III dataset includes several challenges, including missing values, outliers, etc. This may occur due to sensor or transmission failure, error in saving data, etc. Training a model in such noisy and incomplete data is considered the main reason for a model with poor performance. The following subsections discuss the steps taken to handle such data challenges.

#### Irregular time interval

In MIMIC III, vital signs are measured at irregular time. Some of them measured every couple of minutes and other measured every few seconds [82, 83. Unless most ML techniques are not prepared to deal with time series data, some of them could handle it when sampled with the same interval. To solve this problem, we aggregated patient’s vital signs observations to provide a single record every 1 h by taking the average of all measurements over that hour. As a result, each record includes consistent values.

#### Removing outliers

Outliers are values that are too far from the normal range [75, 84]. Normal range specified according to medical expert opinions. The outliers are removed; then, expectation maximization technique is used to impute them [85].

#### Data imputation

Medical data usually include missing values. This returns to several reasons, including sensor failure, recording data at different time intervals, etc. The simple way to handle missing values is to remove them. However, this way may lead to losing significant information. Therefore, several algorithms have been developed to impute missing values based on the other records such as hot-deck encoding [86], and expectation maximization [85]. In the preprocessing stage of MIMIC III, large proportion of data (40–55%) in important features are lost, but we could not eliminate them due to their importance in the prediction process. Considering this issue, we decide to choose cases that have at least 2 values in each measurement, then applying expectation maximization [85, 87] to impute other missing values.

### Feature extraction

In this step, we mainly depend on medical expert opinions in specifying the most important features that could contribute to predict GCS and assure the ability of predicting GCS from vital signs. As mentioned before, GCS is a medical score that used to specify the consciousness of the patient through three main measures: verbal response, eye opening and motor response [88, 89]. These measurements are highly correlated with changing in patient’s vital signs. For example, low blood pressure level may lead to hypotensive; therefore, patient will not be able to respond correctly through verbal or motor response [90]. This is because decreasing blood pressure will result in generating metabolites that cause problem in circulation and tissue functions as well. The same for heart rate, the rapid or slow heart rate will mainly affect the cardiac functions that may cause cardiac arrest or blockage and subsequently affect the circulation. On the other hand, when the heart rate exceeds normal range, it increases the probability of tachyarrhythmia that may be reflected in fibrillation or atrial flutter [91, 92]. Temperature also affects the response of the patients; if the patient has hyperthermia (Temp > 40 C), the temperature autoregulation centers in the brain will be affected [93, 94]. If patient has hypothermia (Temp <  = 35 C), this will affect patient response.

Deceasing in O_2_ saturation may also lead to cardiac arrest or lactic acid accumulation [95]. Low hemoglobin level may also consider a risk indicator, especially with kidney diseases patients, while high level may lead to strokes, clots and heart attacks [91, 96, 97]. There are other features such as PCO_2_, HCO_3_, PO_2_. These features used to specify the percentage of carbon and oxygen in the blood, indicate the level of blood PH and patient’s acid–base balance [98]. All of these are critical situations, affect patient response and may lead to sudden death [99, 100]. Appendix A shows the feature names, Id, normal range and unit of measurement (UoM).

### Results and discussion

The simulation results are carried out on the collected data from the patients using a server with NVIDIA GPU, Intel Core i7 CPU and 32 GB RAM. We used this facility to make sure that the proposed framework can be commonly used in the real application. This work proposes several ML techniques, including Linear Regression (LR), Support Vector Machine (SVM), Decision Tree (DT), k-Nearest Neighbor (*k*-NN) and Ridge Regression (RG). In addition, we deployed some ensemble ML methods, including Random Forest (RF) and Gradient Boosting Regression (GBR). Table 7 illustrates the hyperparameters of each one of the proposed models. These parameters are selected based on several iterations using grid search algorithm in terms of the optimal performance.

**Table 7:** ML algorithms hyperparameters

Algorithm	Coefficient
LR	fit_intercept = True, normalize = True, copy_X = True, n_jobs = -1
SVR	C = 1.0,epsilon = 0.1,kernel = 'rbf'
DT	criterion = 'mse’, splitter = ’best’, max_depth = 20, presort = False
k-NN	n_neighbors = 7, algorithm = ’auto’, leaf_size = 30, p = 2, metric = ’minkowski’
Ridge	alpha = 1.0, fit_intercept = True, normalize = False, copy_X = True,, tol = 0.001, solver = 'auto', random_state = 33
RF	n_estimators = 100, max_depth = 16, random_state = 33
GBR	n_estimators = 100,max_depth = 16, learning_rate = 1.5,random_state = 33

#### Evaluation metrics

The following metrics are used to evaluate the performance of the proposed model, which are Mean Absolute Error (MAE), Mean Square Error (MSE), Median Absolute Error (MedAE) and R^2^ score. These metrics are computed as follows:

1 $${\text{MAE}} = \frac{1}{n} \mathop \sum \limits_{i = 1}^{n} \left| {y_{i} - \hat{y}_{i} } \right|$$

2 $${\text{MSE}} = \frac{1}{n} \mathop \sum \limits_{i = 1}^{n} \left( {y_{i} - \hat{y}_{i} } \right)^{2}$$

3 $${\text{MedAE}} = {\text{median}}\left( {\left| {y_{1} - \hat{y}_{1} } \right|, \left| {y_{2} - \hat{y}_{2} } \right|, \ldots , \left| {y_{n} - \hat{y}_{n} } \right|} \right)$$

4 $$R^{2} = 1 - \frac{{\mathop \sum \nolimits_{i = 1}^{n} \left( {y_{i} - \hat{y}_{i} } \right)^{2} }}{{\mathop \sum \nolimits_{i = 1}^{n} \left( {y_{i} - \overline{y}} \right)^{2} }}$$

where${y}_{i}$ is the ith true value. ${\widehat{y}}_{i}$ is the corresponding predicted value. $n$ is the number of observations. $\overline{y }$ is the mean of the true values.

#### Results without feature selection

**Fig. 5:**
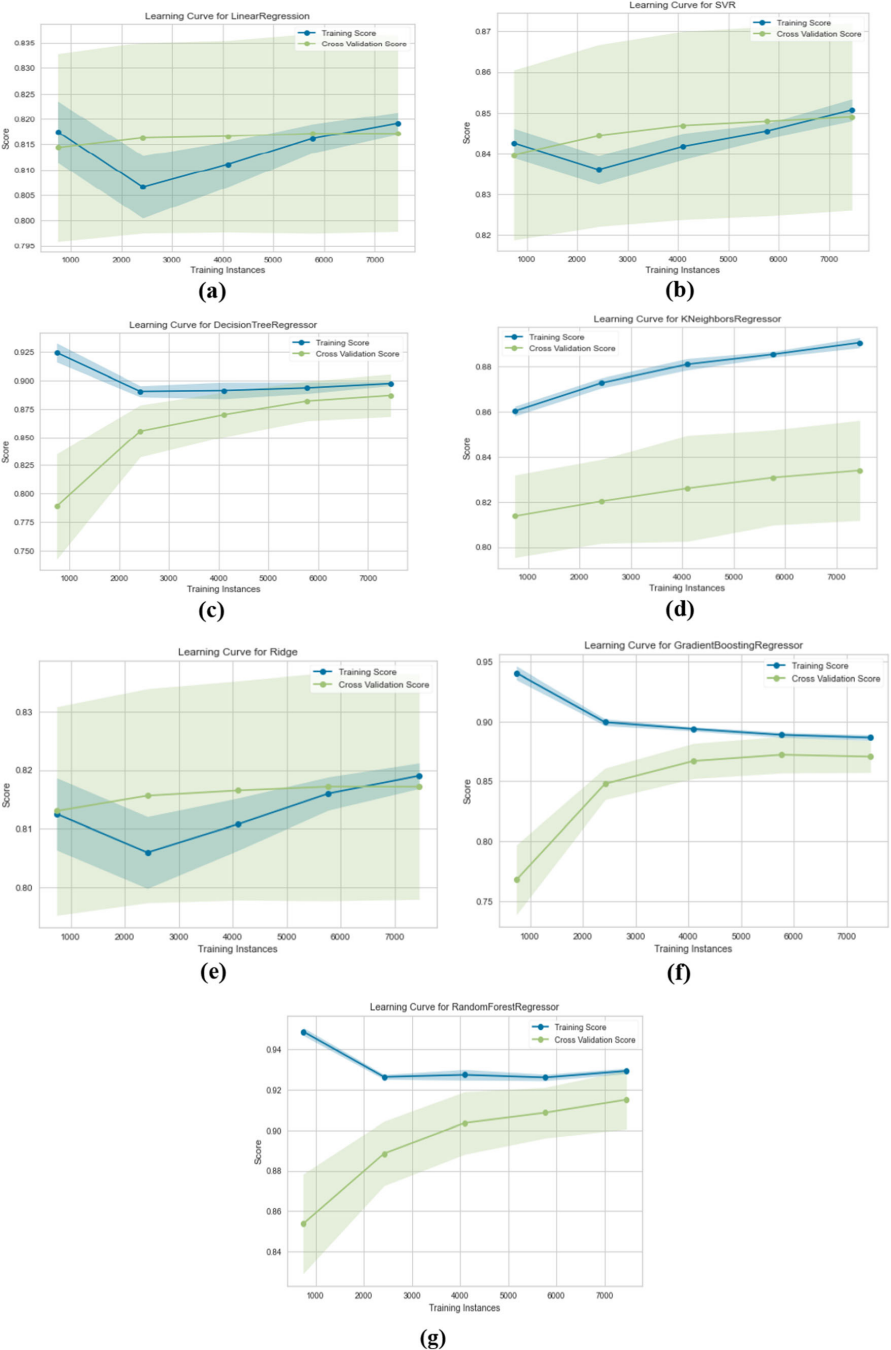
Results of machine learning models without feature selection for both training and testing (**a**) linear regression model, (**b**) support vector regression model, (**c**) decision tree model, (**d**) k-nearest neighbor, (**e**) ridge regression, (**f**) XGBoost, (**g**) random forest regressor

The proposed models are performed on the dataset without feature selection. This scenario is performed to highlight the performance of the proposed models without any preprocessing. Table 8 illustrates the training and testing scores. In addition, it contains the evaluation metrics including MAE, MSE, MedAE and *R*^2^ score. It can be observed that the tree algorithm which is deployed using DT achieved a quite high performance with 0.503, 1.186, 0.348 and 0.8926 for MAE, MSE, MedAE and *R*^2^ score, respectively. Moreover, the ensemble method (RF) appears an optimal performance with 0.406, 0.8385, 0.2839 and 0.9229 for MAE, MSE, MedAE and *R*^2^ score, respectively. Furthermore, Fig. 5a–g shows the learning curves of the proposed methods containing the train and validation curves. It can be observed that the proposed DT and RF methods achieved their optimal performance at number of iterations of 7000, while the other deployed method achieved theirs at 8000. This means that they have a high performance with a low complexity. So, these techniques (DT and RF) can be considered as acceptable automatic GCS prediction solutions without preprocessing. However, there is a need to enhance their performance with some preprocessing like feature selection which is discussed in the next subsection.

**Table 8:** Results of ML models without feature selection

Algorithm	Training score	Testing score	MAE	MSE	MedAE	*R*^2^ score
LR	0.819	0.816	0.701	1.995	0.381	0.816
SVM	0.850	0.849	0.3926	1.642	0.08591	0.8491
**DT**	**0.897**	**0.896**	**0.503**	**1.168**	**0.348**	**0.8926**
KNN	0.891	0.835	0.570	1.795	0.119	0.835
Ridge	0.819	0.816	0.711	1.993	0.392	0.816
(GBR)	0.885	0.880	0.572	1.3031	0.2578	0.880
**RF**	**0.929**	**0.922**	**0.406**	**0.8385**	**0.2839**	**0.9229**

#### Results with feature selection

**Fig. 6:**
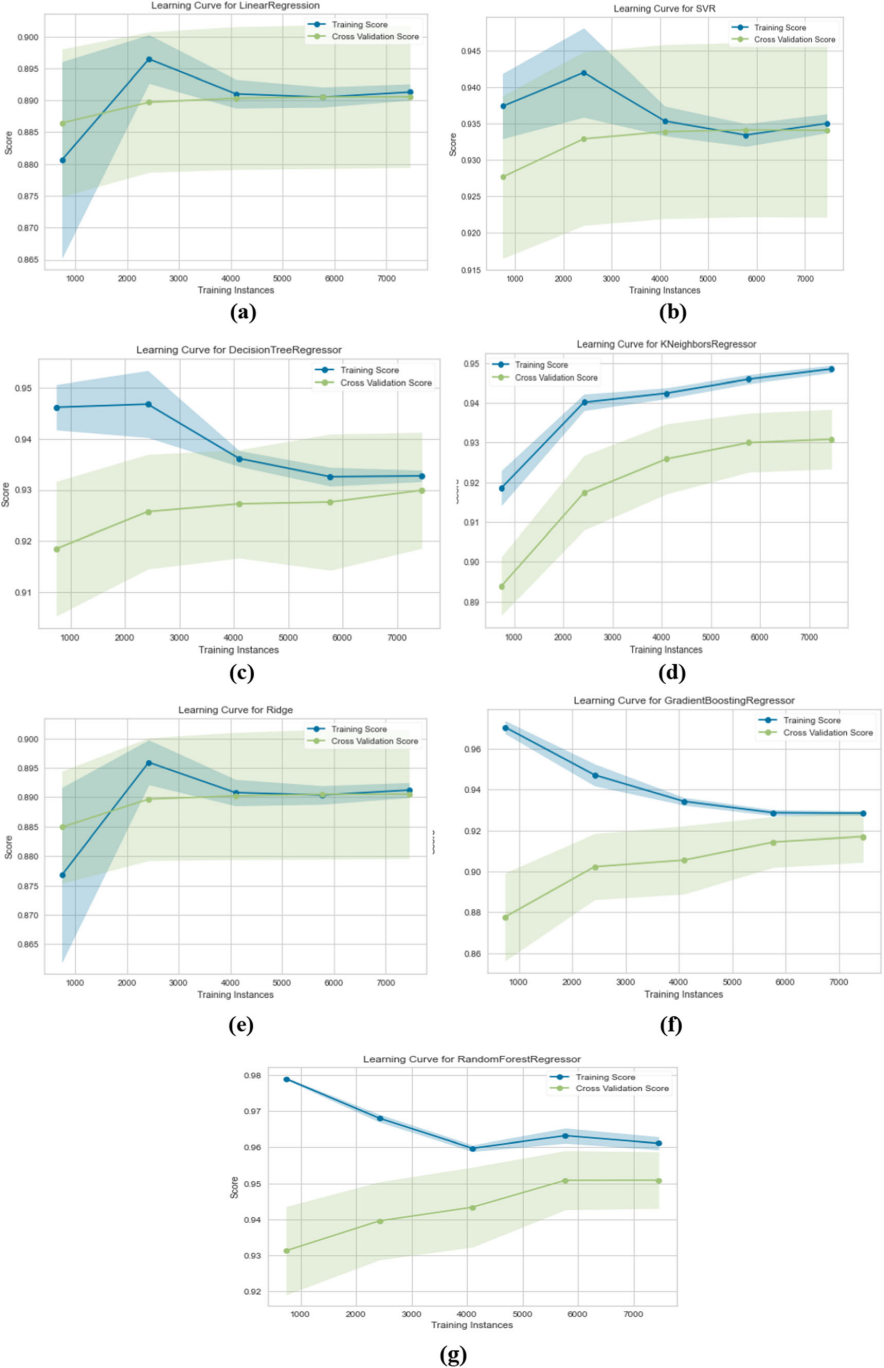
Results of machine learning models with feature selection for both training and testing **(a)** linear regression model, (**b**) support vector regression model, (**c**) decision tree model, (**d**) k-nearest neighbor, (**e**) ridge regression, (**f**) XGBoost, (**g**) random forest regressor

In this section, we perform feature selection algorithm which utilized to magnify the impact of the input features by selecting the most important features to estimate the output value. For this purpose, we used the recursive feature elimination (RFE) algorithm with feature scaling to figure out the most impacted feature by a recursive elimination process which leads to a high performance. Table 9 illustrates the simulation results of the proposed models. It can be observed that DT model achieved 0.39, 0.961, 0.25 and 0.917 for MAE, MSE, MedAE and *R*^2^ score, respectively. In addition, RF model achieved 0.269, 0.625, 0.0784 and 0.946 for MAE, MSE, MedAE and *R*^2^ score, respectively. Thus, it can be noticed the performance of both DT and RF model is considerably increased by deploying feature selection. Furthermore, the proposed SVM regressor achieved 0.283, 0.813, 0.0844 and 0.929 for MAE, MSE MedAE and *R*^2^ score, respectively. So, the proposed models can be considered as efficient solutions for detection of level of consciousness. Figure 6a–g shows the training and testing performance of the developed model performance.

**Table 9:** Results of ML models with feature selection

Algorithm	Training score	Testing score	MAE	MSE	MedAE	*R*^2^ score
LR	0.8912	0.888	0.652	1.304	0.408	0.888
**SVM**	**0.934**	**0.929**	**0.283**	**0.813**	**0.0844**	**0.929**
**DT**	**0.932**	**0.917**	**0.390**	**0.961**	**0.25**	**0.917**
KNN	0.949	0.934	0.350	0.762	0.0	0.934
Ridge	0.8911	0.885	0.658	1.305	0.414	0.888
(GBR)	0.927	0.916	0.475	0.974	0.240	0.916
**RF**	**0.960**	**0.940**	**0.269**	**0.625**	**0.0784**	**0.946**

### Model explainability

ML and deep learning (DL) have been widely used in predicting and diagnosing various diseases such as predicting hypertension [101], diabetes [102], sepsis [103]. Unfortunately, most of these studies concentrated on achieving advances in the overall performance of the developed models, while disregarding the interpretability issues. ML and DL are considered as a black box that is unable to provide answers for the medical straightforward inquiries (i.e., why it developed this decision, what the correlation between patient’s medical features and the developed output, etc.). Therefore, the physicians find it complex to depend on such models without a clear and understandable explanation. As a result, there exists a serious gap between the developed models and their utilization in medical practice.

In the latest years, a quite number of studies tried to justifying this issue by explaining the developed models using what is known as explainable artificial intelligence [73, 74, 104]. Explainable artificial intelligence or explainability (XAI) is the ability of ML and DL models to open the black box and provide natural language explanation for the developed decisions [105–107], explain what occurred in the developed model from input features to the final output. It utilized to help non-ML experts to understand the solutions developed by ML models. Therefore, we not only concentrate in developing ML model that could predict GCS based on vital signs and laboratory tests, but also provide an explanation for the developed decision. In this work, we depend on the SHAP library in the explanations issue [108]. SHAP explainers usually depend on a tree-based classifier (i.e., DT, RF, XGBoost, LightGBM, etc.) to calculate the contribution of each feature in the decision [109, 110]. Furthermore, we utilize the internal logic of the RF regressor to discuss the explainability of features as well as cases.

#### Explainability of features (globally)

Feature importance gives a general view of the rank of all features and the impact of each feature on the final decision. In this work, we depend on RF to calculate the importance of all features. Table 10 shows the feature importance for all features. Unfortunately, we cannot depend on it to specify the direction of each feature. For instance, we cannot specify if increasing the cardiac index will contribute to increasing the overall score of consciousness or not.

**Table 10:** Importance of features according to RF model

Feature name	Correlation using RF
Heart rate	0.09850
SpO_2_	0.09146
Temperature C (calc)	0.04965
Arterial blood pressure systolic	0.03043
CVP alarm [low]	0.04972
O_2_ flow	0.02934
CVP alarm [high]	0.02933
Respiratory rate	0.08911
HCO_3_ (serum)	0.02906
Hemoglobin	0.02817
Blood urea nitrogen (BUN)	0.07517
Creatine kinesis	0.06293
WBC	0.02269
Insulin	0.02019
Cardiac index	0.01621
Cholesterol, HDL	0.0349
Respiratory effort	0.06302
Arterial blood pressure diastolic	0.06255
Glucose finger stick	0.05606
Arterial pH	0.01021
weight	0.01822
Albumin	0.02703
Urine out void	0.00257

**Fig. 7:**
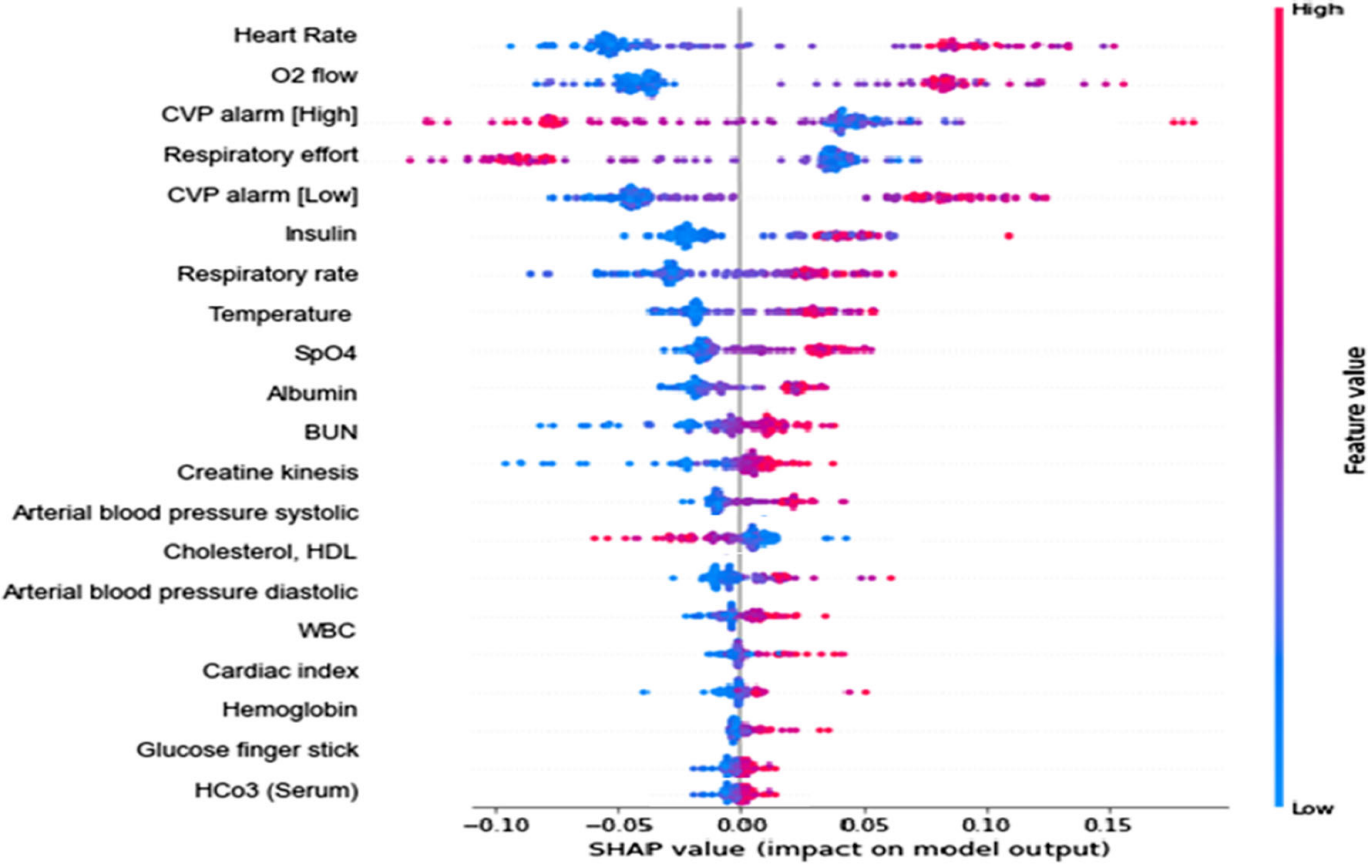
SHAP summary plots for the proposed model

In this section, we utilized SHAP summary plots to show the rank of each feature. As shown in Fig. 7, each line represents one feature, and each dot represents the effect of this feature in a specific instance. Feature correlation is represented by colors (blue for low correlation, and red for high correlation). From the summary plot, we can observe the following: (1) heart rate has a significant impact on the overall decision. (2) Increasing heart rate and O_2_ flow value have a positive impact in increasing the overall score. (3) On the contrary, decreasing the value of cholesterol, respiratory effort and CVP alarm [high] have a positive impact on the overall performance of the calculated score. (4) Summary plot also allows us to specify the impact of the outliers. For example, the respiratory effort is not the global critical feature, but it has a high negative effect on some cases. This is indicated in the long-tail that is distributed along the left direction. Features that have a long tail in the right direction are likely to have a positive effect on the total decision. Our medical expert reported that this is a medically intuitive issue that increases the confidence in our model.

To ensure the impact of the chosen feature in the developed model, we extract the feature importance using RF model. Table 10 illustrates the correlation among the whole features and the estimated output. It can observe that there are some features have high correlation such as heart rate, temperature and blood pressure. Others have low correlation values such as cardiac index and arterial PH.

#### Explainability of cases (locally)

**Fig. 8:**
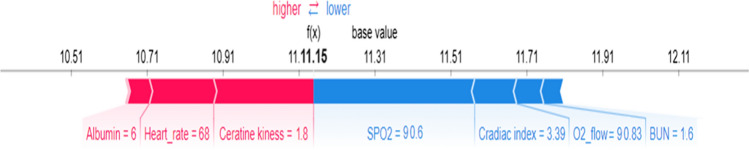
SHAP model behavior for cases

In this section, we will discuss the explainability for each case. For example, as we show in Fig. 8, each example represents a horizontal line. It firstly shows the final prediction for this case (GCS = 11.15). It also shows the features that have a positive impact on the final decision (heart rate = 68, albumin = 6), and features that push the total prediction away from the optimal values (SpO_2_ = 90.6%, O2_flow = 90.83% and BUN = 1.6). This represents the effect of each feature in the final output by colors (red = supported, blue = not supported).

## Discussion

### Comparison between model performance before and after feature selection (FS)

**Fig. 9:**
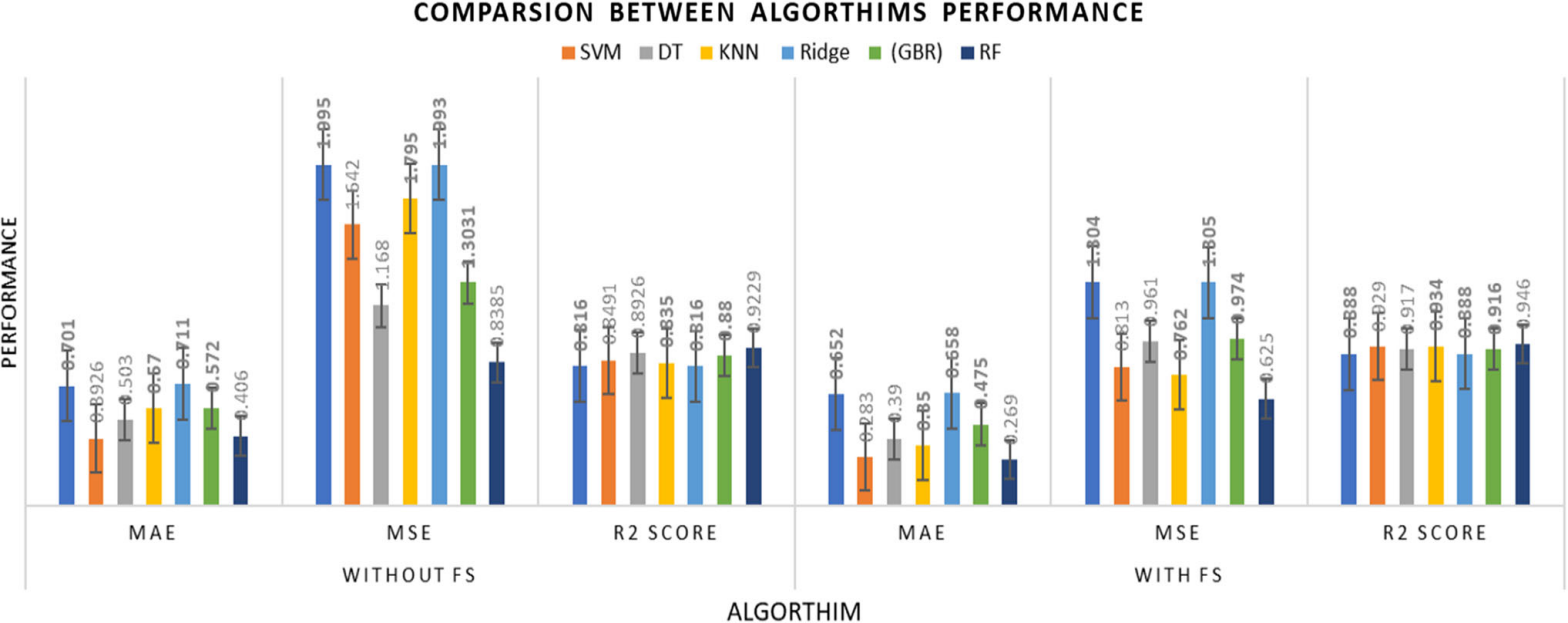
Comparison between models performance (before and after FS step)

**Fig. 10:**
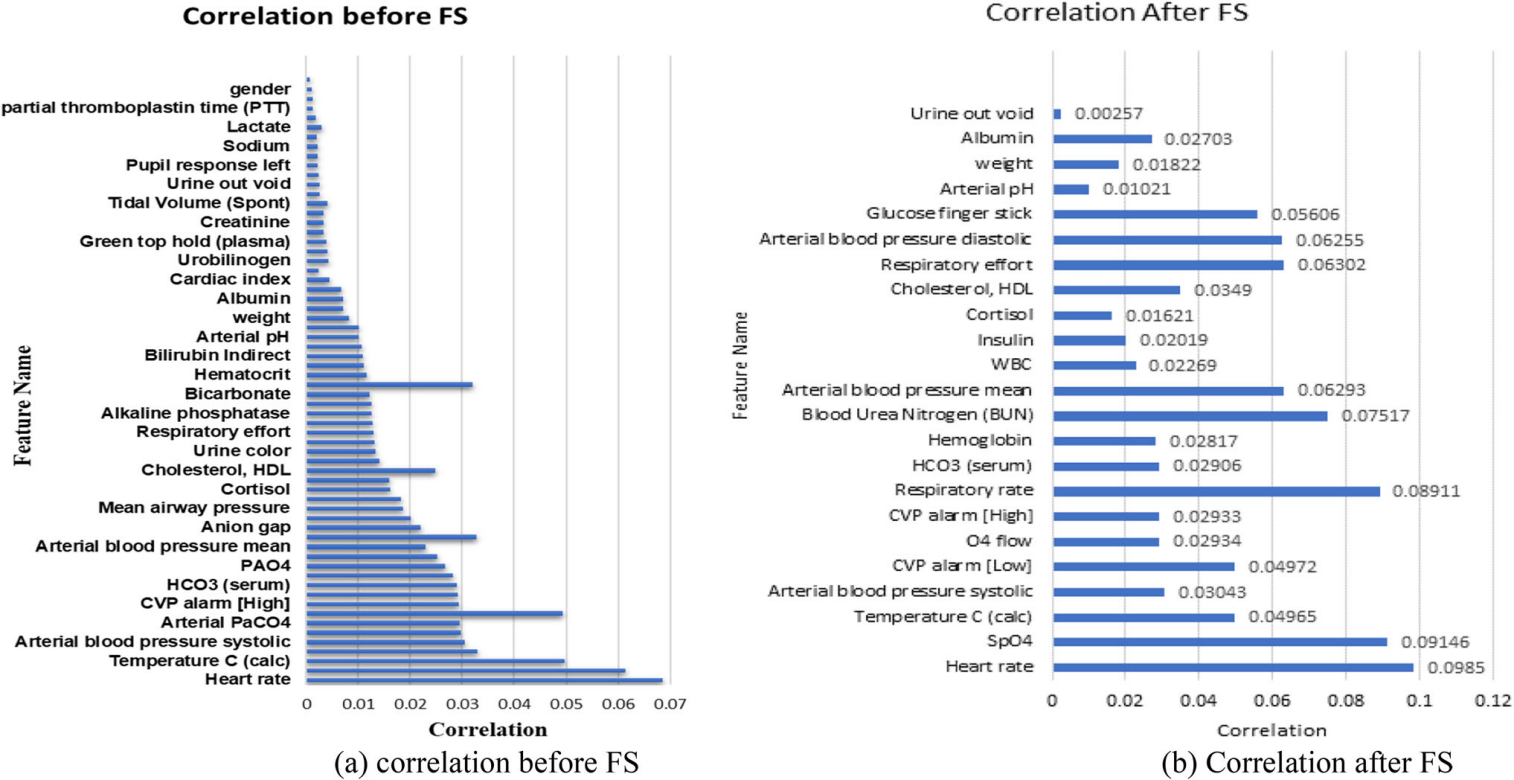
**a** Correlation before FS, **b** Correlation after FS

In this section, we compare the performance of our proposed model in terms of the two-feature list (before and after feature selection). Figure 9 shows comparison between all models in terms of MAE, MSE and R^2^ score. From this figure we can observe that the performance improved about 2–6% for all evaluation metrics. This enhancement ensures the importance of this step in the final result. Figure 10a, b shows correlation between the feature list and the output before and after FS stage.

### Study limitations

Although our proposed model provides a promising solution for GCS automatic calculation, it still has some limitations that need to be handled. *First*, there are some situations in which changes in vital signs will not affect GCS, such as small changes in blood pressure that did not reach hypotension, hypothermia that did not affect thermoregulation center. *Second*, MIMIC dataset was extracted from one institute; therefore, using MIMIC dataset to evaluate the developed model does not guarantee the generalization ability of the model. *Third,* the imputing process for several important features could negatively affect model performance. Therefore, we intend to investigate several imputation techniques. All of these limitations will be addressed in the future studies.

### Comparison with the works in the literature

This paper proposes a GCS prediction system to estimate the level of consciousness of the patients based on their vital signs. For this purpose, several machine learning techniques are deployed to achieve the optimal method. The simulation results are carried out on the MIMIC III dataset with the interest of the vital signs and the level of consciousness. This paper proposes several machine learning models to handle this issue. The simulation results are carried out on the data with and without feature selection. The feature selection is performed based on the importance of the features and their impact on the output according to their correlation with the output. The simulation results reveal that the proposed SVM, KNN, DT and RF models achieved the optimal performance prior to GCS value prediction. To highlight the performance of the proposed system, we illustrate the impact of the proposed system with the works in the literature. The objective of the proposed system is to predict the value of the GCS using regression. The works in the literature focused on the classification as a solution for this issue. As proposed in [4], they categorized the GCS into three ranges and performed machine learning techniques to classify among them. We show the performance of the proposed system with the proposed in [4] in terms of the mutual machine learning techniques, including KNN, SVM and RF. We compare their performance as classifiers and regressors to solve the problem of diagnosis of level of consciousness. The regression method is evaluated by *R*^2^ score, while the classification method is evaluated by accuracy. Both of the evaluation metrics are within range of 0–1. As shown in Table 11, the proposed models without feature selection achieve 0.835, 0.849 and 0.923 for KNN, SVM and RF, respectively. On the other hand, these models with feature selection achieved 0.934, 0.929 and 0.946 for KNN, SVM and RF, respectively. Therefore, it can be observed that the regression trend achieved a quite high performance rather than classification trend prior to diagnosis of level of consciousness.

**Table 11:** Illustration of the proposed work and the works in the relation

Work	Method	Model	Metric	Performance
Proposed	Regression	KNN	*R*^2^ score	0.835
		SVM		0.894
		RF		0.923
		KNN + feature selection		0.934
		SVM + feature selection		0.929
		RF + feature selection		0.946
[2]	Classification	KNN	Accuracy	0.875
		SVM		0.831
		RF		0.925

## Conclusion

The problem of detection of level of consciousness has been discussed in this work. This issue has been handled in the presence of IoT system and cloud/edge environment. The proposed framework is based on deploying machine learning for automatic prediction of the level of consciousness based on some vital signs and laboratory tests. Therefore, several machine learning models including both ensemble and kernel models have been implemented to provide a judgeable comparison and extensive study. The simulation results reveal that the proposed ensemble models present a superior performance prior to prediction of the level of consciousness. Therefore, it can be considered as an efficient solution for consciousness level prediction in IoT and cloud/edge environments.

## Data Availability

Data are available on request due to ethical restrictions.

## Appendix A: Features description

**Table:**

Item_ID	Label	Normal range	Unit of measurement
619, 224,690	Respiratory rate	12–16	Breaths per minute
2381, 220,045	Heart rate	60–100	Beats per minute
646, 5820	SpO_2_	> 90	%
470	O_2_ flow	80–100	%
1525	Creatine	0.74–1.35	mg/dL
3066,772	Albumin	0.4–5.4	g/dL
116, 7610	Cardiac index	2.5–4.0	l/min/m^2^
1162,781	Blood urea nitrogen (BUN)	6–24	mg/dL
220,050, 220,179	Blood pressure	120/80	mmHg
676, 223,762	Temperature	36.1–37.2	Celsius
440,546, 227,062	WBC	(4–11)000	N/μL
225,664	Glucose finger stick	80–130	mg/dL
	Insulin	80–130	mg/dL
50,909	Cortisol	6–8 am,10–40 pm	mcg/dL
50,904	Cholesterol, HDL	40–59	mg/dL
616	Respiratory effort	–	–
780	Arterial pH	7.25–7.55	mmHg
40,069	Urine out void	0.3–0.5 ml for kg per H	mL
	Weight		Kg
	Age		Y
198, 227,015	GCS	11–15	

(mg/dl) milligrams/deciliter

(g/dl) grams/deciliter

## Appendix B: List of abbreviations

**Table:**

Term	Abbreviations
DL	Deep learning
LR	Linear regression
SVR	Support vector regression
KNN	K nearest neighbor
MLP	Multilayer perceptron
RF	Random forest
BUN	Blood urea nitrogen
WBC	White blood cells
GCS	Glasgow coma scale
UoM	Unit of measurement
XAI	Explainability artificial intelligence
SHAP	Shapley additive explanations
SDN	Software defined network
IoT	Internet of things
PCA	Principle component analysis
FACM	Fog-Assisted Consciousness Management
MAE	Mean absolute error
MSE	Mean square error
